# Antiviral Therapy of Chronic Hepatitis B Virus between Present and Future

**DOI:** 10.3390/jcm13072055

**Published:** 2024-04-02

**Authors:** Mariana Daniela Ignat, Alexia Anastasia Stefania Balta, Raisa Eloise Barbu, Miruna Luminita Draganescu, Luiza Nechita, Doina Carina Voinescu, Aurel Nechita, Ioana Anca Stefanopol, Camelia Busila, Liliana Baroiu

**Affiliations:** 1 Doctoral School of Biomedical Sciences, ‘Dunarea de Jos’ University, 800008 Galati, Romania; mariana_daniela52@yahoo.com (M.D.I.); raisauibariu@gmail.com (R.E.B.); 2 Clinical Medical Department, Faculty of Medicine and Pharmacy, ‘Dunarea de Jos’ University, 800008 Galati, Romania; miruna.draganescu@ugal.ro (M.L.D.); nechitaluiza2012@yahoo.com (L.N.); doina.voinescu@ugal.ro (D.C.V.); aurel.nechita@ugal.ro (A.N.); camelia.busila@ugal.ro (C.B.); liliana.baroiu@ugal.ro (L.B.); 3 ‘Sf. Cuv. Parascheva’ Clinical Hospital of Infectious Diseases, 800179 Galati, Romania; 4 ‘Sf. Apostol Andrei’ Clinical Emergency County Hospital, 800578 Galati, Romania; 5 ‘Sf. Ioan’ Clinical Hospital for Children, 800487 Galati, Romania; anca.stefanopol@ugal.ro; 6 Clinical Surgical Department, Faculty of Medicine and Pharmacy, ‘Dunarea de Jos’ University, 800008 Galati, Romania

**Keywords:** hepatitis B virus, functional cure, antivirals

## Abstract

**Background/Objectives**: The objective of this study was to analyze the results of clinical trials regarding long-term antiviral therapies in chronic hepatitis with HBV to compare current therapeutic protocols and to analyze the results of preliminary studies with new antiviral therapies for HBV. **Methods**: Clinical studies and meta-analyses from PubMed, Google Scholar, and Research Gate from 2011 to 2024 were analyzed on patients undergoing chronic antiviral therapy for HBV, and a retrospective observational study performed in our clinic on a group of 76 patients undergoing chronic therapy with entecavir was presented. Also, a summary of the results of preliminary studies with various innovative antiviral molecules for HBV was performed. **Results**: The results of extensive clinical trials reveal that current therapies for chronic HBV are well tolerated and maintain good viral suppression if the patient is adherent to therapy. Innovative therapies aim to eliminate HBsAg and, thus, significantly shorten the duration of treatment, and the preliminary results of the studies are promising. **Conclusions**: Being an asymptomatic condition that requires life-long therapy, adherence to therapy is a real problem. Also, the risk of decompensation of liver cirrhosis and adenocarcinoma remains important in these patients. Future research is needed to perfect some antiviral therapy schemes that shorten the treatment period but also decrease the rate of progression towards decompensated cirrhosis and liver adenocarcinoma.

## 1. Introduction

Chronic viral hepatitis B is considered to be a public health problem with great significance. Approximately 1,500,000 individuals positive for hepatitis B virus infection are identified each year and, according to the World Health Organization, in 2019, approximately 296,000,000 people were infected with hepatitis B virus (HBV) globally [1]. The risk of complications such as liver cirrhosis, hepatocellular carcinoma, and hepatic decompensation in individuals infected with HBV [2] is greater than those in the general population. Thus, approximately 25% of liver cirrhosis cases and 30% of hepatocellular carcinoma cases are caused by HBV [3]. Due to these complications, 820,000 individuals died in 2019 as a result of HBV infection [1]. Therefore, chronic hepatitis B is the seventh leading cause of death worldwide [4,5,6]. The prevalence varies globally. For example, the United States of America and Western Europe have a low prevalence. Intermediate prevalence is found in Japan and Mediterranean countries. A high prevalence is found in Southeast Asia and Sub-Saharan Africa. Among the Chinese population, approximately 5 to 6% are HBV-positive individuals [7,8].

The current therapy options for HBV infection aim to decrease the number of new HBV infections and reduce the risk of developing complications, therefore improving these patients’ quality of life. The options consist mainly of vaccination and nucleos(t)ide analog (NA) therapy [9]. Antiviral therapy is initiated after a thorough assessment that includes gauging the stage of liver damage, viral activity, and replication [10,11]. 

Thus, in areas where primary medical care is deficient, there is limited access to testing and treatment, which leads to a high number of new cases and decreased antiviral therapy adherence [10]. 

The research for novel and superior antiviral therapies is ongoing, and the medical scientific communities are doing their best in order to control the HBV infection [9].

Functional recovery, stabilization of the parenchymal lesion, and elimination of HBV are the goals of optimal therapy in chronic hepatitis B [2,12]. Approximately 1% of untreated patients spontaneously lose hepatitis B surface antigens (HBsAgs) yearly [8,13]. Only 1 to 13% of patients treated with NAs and 3 to 11% of patients treated with interferon-alpha (IFN-α) can lose HBsAgs [8,14].

The purpose of this study was to analyze the results of clinical trials regarding long-term antiviral therapies in chronic hepatitis with HBV, to compare current therapeutic protocols, and to analyze the results of preliminary studies with new antiviral therapies for HBV.

## 2. Materials and Methods

We conducted searches in the databases PubMed, Google Scholar, and Research Gate and examined meta-analyses and clinical studies from 2011 to 2024, using keywords such as HBV, treatment, antiviral therapies, clinical studies, entecavir, and tenofovir. The inclusion criteria were studies that included patients with chronic HBV infection for over 6 months, patients not previously treated with antivirals other than entecavir and tenofovir, studies that used antivirals simultaneously, studies with patients not undergoing immunosuppressive treatment, studies with full-text availability, and a minimum of 50 patients included in each study.

The exclusion criteria were studies that include patients with HCV, HDV, and HIV coinfection; alcoholic liver disease and fatty liver; and patients under 18 years old. Out of the total 1230 articles found on PubMed, after excluding articles that did not meet the inclusion criteria, we were left with five clinical studies to which we added unpublished results from a study conducted at the Infectious Diseases Clinical of the St. Cuv. Parascheva Infectious Diseases Clinical Hospital, Galati. This study was a retrospective observational clinical study carried out between 1 November 2022 and 29 February 2024. The study included patients with chronic HBV who agreed to participate in the study, aged over 18 years old, with chronic treatment with entecavir for at least 6 months, established based on the national criteria in Romania for initiation of antiviral therapy (HBV DNA with over 2000 IU, minimum F1 fibrosis or A1 inflammation on FibroMax, or over 7 kPa on FibroScan or liver elastography; HBV-positive DNA regardless of value for F4 on FibroMax, or over 11.7 kPa on FibroScan or liver elastography; and patients undergoing immunosuppressive therapies before transplant procedures). The exclusion criteria of patients from this study carried out in our clinic were refusal to participate in the study; undergoing antiviral therapy for less than 6 months; age under 18 years; death during the study period; extreme values in the studied analyses; and patients with HIV, HCV, and HDV co-infection. This study was approved by the Ethics Council of the St. Cuv. Parascheva Infectious Diseases Clinical Hospital, Galati, with the number 11682, dated 13 October 2022.

## 3. Results

### 3.1. Current Practices Regarding Hepatitis B Infection-Specific Therapy

According to the American Association for the Study of Liver Diseases (AASLD) guidelines, approved antiviral medications for the treatment of chronic hepatitis B include nucleotide analogs (NAs), conventional IFN-α, and Peg Interferon-α (Peg-IFN-α). NA includes lamivudine (LAM), adefovir dipivoxil (ADV), telbivudine (LdT), entecavir (ETV), and tenofovir disoproxil fumarate (TDF). There is a significant success rate of the hepatitis B envelope antigen (HBeAg) seroconversion by using Peg-IFN-α therapy [15,16,17,18]. Furthermore, a new nucleotide analog, tenofovir alafenamide (TAF), was approved in 2016 [11].

The international consensus uses nucleotide analogs (NAs) as first-line therapeutic agents [19]. These reverse-transcriptase inhibitors have a direct effect on HBV polymerase, which they inhibit. The DNA chain becomes elongated and changes the reverse transcription process of DNA.

TAF presents a more stable form than TDF in plasma. By choosing TAF in the patient’s treatment plan, we can decrease the risk of nephrotoxicity and bone toxicity [11] due to TAF’s increased metabolic action inside the hepatocytes, allowing for the use of lower doses.

Regarding the criteria for initiating antiviral therapy, the European Association for the Study of the Liver (EASL), the AASLD, and the Asian Pacific Association for the Study of the Liver (APASL) have similar guidelines. It is recommended that patients’ evaluation should comprise:

- HBsAg;
- HBeAg;
- Viral load of HBV (HBV DNA);
- Hepatic fibrosis and inflammation;
- Laboratory tests.

Even though hepatic biopsy can be performed in order to determine the stages of liver fibrosis and inflammation, it counts as an invasive method, frequently refused by patients. That is why the evaluation contains non-invasive methods, such as FibroScan or hepatic transient elastography, which measure the degree of fibrosis, and FibroMax for both fibrosis staging and liver inflammation grading [15]. 

Regarding the laboratory tests that are recommended, the most important is ALT—a biochemical marker that reflects the degree of liver damage, liver inflammation, and viral activity [15].

The presence of severe comorbidities, such as the need to institute an immunosuppressive treatment, an organ transplant, or the presence of liver cirrhosis requires starting the therapy with nucleotide analogs as quickly as possible, regardless of the degree of liver fibrosis.

Entecavir is a cyclo-pentanol–guanosine analog that has strong anti-HBV activity and rapidly reduces viral load in patients with chronic HBV. In two Phase III clinical trials in HBeAg-negative and HBeAg-positive patients, after 48 weeks of treatment with entecavir, it was observed that, respectively, 90% and 67% achieved undetectable HBV DNA, 78% and 68% had ALT levels within the normal range, and 0 and 2% lost HBsAg [20,21]. Among HBeAg-positive patients with 5 years of ETV treatment, 94% achieved HBV DNA < 300 copies/mL, 80% showed normal ALT values, and 5% achieved HBsAg loss [22]. Entecavir, an oral deoxy-guanosine nucleoside analog, is highly active against HBV and has a high genetic barrier to resistance [23].

Tenofovir disoproxil fumarate is a nucleotide analog that inhibits HBV polymerase. In two Phase III clinical trials in HBeAg-negative and HBeAg-positive patients treated with TDF for 8 years, respectively, 99% and 98% had undetectable HBV DNA, while 88% and 84% achieved ALT normalization. Meanwhile, 1.1% and 13% experienced HBsAg loss [24].

Tenofovir alafenamide, a pro-drug of tenofovir, delivers active metabolites to hepatocytes more efficiently than TDF. In two Phase III clinical trials, for HBeAg-negative and HBeAg-positive patients, after 3 years of TAF treatment, respectively, 87% and 74% achieved HBV DNA levels below 29 IU/mL, while 71% and 64% had normalized ALT. Meanwhile, 0.4% and 1.4% achieved HBsAg loss [25,26].

Most of the research on the TDF-to-TAF switch comes from studies involving HIV/HBV-coinfected patients. In studies lasting up to 96 weeks, switching to TAF compared to continuing TDF (as part of an antiretroviral regimen) has been associated with improvements in proteinuria, albuminuria, and proximal tubule function (especially in the first 24 weeks), as well as bone mineral density [10,27]. Overall, these studies have shown that TAF has a better safety profile than TDF and similar antiviral efficacy in studies lasting up to 2 years [10].

NAs have a reportedly strong antiviral effect in patients with liver cirrhosis caused by HBV. However, the rate of HBsAg loss in patients is low [8].

IFNα has multiple actions (antiviral, immunomodulatory, antiproliferative), which is why treatment, including IFNα, needs a shorter time period to achieve increased virologic response and earlier loss of HBsAg compared to nucleos(t)ide analogs [28,29]. The downside of using IFNα is the many dose-dependent side effects.

### 3.2. Limitations of Current Antiviral Therapy

In the current timeframe, many choose NAs over α-PEG-IFN due to their favorable adherence, strong antiviral activity, better safety profile, and lower antiviral resistance [30]. NA therapy has its long-term drawbacks—specifically, the low rates of seroconversion of AgHBs and AgHBe [31] and the lack of direct action over covalently closed circular DNA (cccDNA) activity. Some levels of cccDNA could still be detected in the infected liver despite therapeutic success. To sum up, NA treatment is considered a long-term approach [32,33].

### 3.3. Pharmaceutical Resources to Prevent HBV Infection

- Passive immunization with anti-HBV immunoglobulins is indicated, according to the Romanian protocol, immediately after the accident (contact with human fluids infected with HBV) or immediately after birth in children of HBV-infected mothers [34].
- Active immunization with HBV vaccine is recommended for all newborns as soon as possible after birth (within 24 h), followed by two or three doses at least four weeks apart [35]. The duration of vaccine protection is at least 20 years and probably for life [36]. It is also recommended to vaccinate with three doses (0, 1, 6 months) in population groups at risk: people who frequently require blood and/or blood products, dialysis patients, solid organ transplant recipients, persons institutionalized in prisons, intravenous drug users, persons with domestic and sexual contact with persons with chronic HBV infection, people with multiple sexual partners, healthcare workers and others who are exposed to blood or blood products through work, and travel to endemic areas without the complete hepatitis B vaccination [36,37].

A booster dose is given to populations at risk in case of anti-HBs < 10 mIU/mL [35].

The impact of universal HBV immunization is huge, with 90–95% efficacy in preventing chronic HBV infection and ~70% protective efficacy against hepatocellular carcinoma [36,38].

- Antiviral medication, such as tenofovir, is indicated in the prophylaxis of vertical transmission in HBsAg-positive mothers with high viremia (HBV DNA > 200,000 IU/mL) and/or HBsAg > 4 log 10 IU/mL, administered in the last trimester of pregnancy, and to mothers on antiviral treatment at the beginning of pregnancy, where it is administered throughout the pregnancy [34,39].

### 3.4. Innovative Therapies Approved for Treatment and under Development

#### 3.4.1. Direct-Acting Antivirals (DAAs)

HBIG (hepatitis B immunoglobulin) is an antiviral agent that belongs to the class of polyclonal antibodies neutralizing HBsAg [40]. It has a direct action on the antigenic loop (AGL) present in the S domain and a neutralizing and binding action over the circulating virions which prevents hepatocyte infection. The main concern regarding this type of therapy revolves around the very high cost determined by the limited availability (the production process is difficult because the molecules are exclusively obtained from vaccinated donors), a very long processing duration, and a lack of efficacy against AGL mutations in the S domain [41].

In the idea of compensating for these shortcomings, monoclonal antibodies that act on the preS1 domain have been created and they present promising results in the preclinical experiments [42,43].

There are certain cases where the use of antiviral therapy is limited—for example, preventing reinfection of the liver graft in transplant patients or preventing vertical transmission from mother to fetus [44,45].

GC1102 is part of the class of monoclonal antibodies that neutralize AgHBs and is currently in Phase II of development [46].

HzKR359-1, HzKR127-3.2 belong to the class of monoclonal antibodies acting on the preS1-level and are in the preclinical stage of development [41].

In a study conducted by Wi J et al. [41], a humanized version of (KR359)—HzKR359-1 was developed, whose antigen-binding capacity was measured using ELISA technique and it is 4.4 times higher than that of the KR359 molecule. It produces recombinant preS1 antigens for various genotypes to investigate the binding ability of HzKR359-1 and a humanized version HzKR127-3.2 for the 10 genotypes of HBV.

The antibodies bind as follows: both HzKR359-1 and HzKR127-3.2 bind to genotypes A and C (common genotype); HzKR359-1 can bind to genotypes B, H, and J, (genotype B is also a common one); HzKR127-3.2 can also bind to genotypes D, G, and I (genotype D is also frequently identified).

Developing a therapeutic regime with these combined antibodies should block the entry of viruses with these genotypes into the cell, thus preventing HBV infection [41].

Heparin, Suramin, and synthetic anti-lipopolysaccharide peptides (SALPs) inhibit the interaction between HBV and HSPG (heparansulfate proteoglycan) and are in the preclinical stage. Heparin and suramin are artificial or natural derivatives of HSPG, which can inhibit HBV infection [40]. SALPs are a new class of peptides that inhibit infection by a wide range of viruses by binding to heparansulfate fractions on the cell surface [47]. The principle of action is based on the ability to bind to lipopolysaccharide peptides (LPS), the Limulus anti-LPS factor (LALF) [48], which neutralizes LPS and thus blocks both in vitro and in vivo immunological consequences [49].

PAC (proanthocyanidine) and its analogs act on the Anti-preS1 oligomeric flavonoidanalog level and are in the preclinical stage. According to Tsukuda [50], based on cell chemical screening, it was found that PAC inhibits HBV infection, and the cytotoxic effect is minimal. PAC acts by preventing the attachment of preS1 region of the large surface protein (LHB) to the specific sodium taurocholate cotransporting polypeptide (NTCP) receptor. Additionally, the anti-HBV activity of the PAC molecule was pan-genotypic, against an isolated nucleoside HBV infection, and increased the capacity of the nucleoside analog (tenofovir).

Conjugated bile acids (TCA, UDCA, TUDCA), Ezetimibe, Irbesartan, Cyclosporine analogs (SCY450, SCY995), and Bulevirtide belong to the class of NTCP-inhibitors, entry inhibitors for HBV and HDV.

#### 3.4.2. Antivirals That Act on the cccDNA

This class includes Interferons, tumor necrosis factor alpha (TNF-α), and agonists of lymphotoxin β-receptors. In the study conducted by Xia et al. [51], several monocytic mechanisms that can eliminate HBV through T-cells from hepatocytes were investigated. Among the methods used by Xia et al., we find enzyme-linked immunosorbent assay, in situ hybridization analysis of liver biopsies, as well as incubation with interferon-γ or TNF-α, or through co-cultivation of T-cells, HepG2-H1.3 cells, VHB-infected HepaRG cells, and primary human hepatocytes. Additionally, cccDNA and viral replication markers were measured. In conclusion, it was observed that both IFNγ and TNF-α reduce cccDNA levels through deamination and subsequent disintegration of cccDNA [51].

#### 3.4.3. Antivirals That Act on Various Stages of the HBV Life Cycle

Entry inhibitors, such as Bulevirtide, block the binding of HBV to the NTCP receptor. Additionally, in a Phase IIb clinical trial, 9 out of 30 patients achieved a >1log10 decrease in HBsAg levels following the administration of Bulevirtide and PEG IFN-α [8,52]. Moreover, in the monotherapy group, no change in HBsAg levels was observed. Bulevirtide is well-tolerated by patients, but it has limited effects on HBsAg [52]. Nevertheless, it represents an important step in the treatment of HDV.

Antivirals that act on viral transcription: JNJ-3989, VIR-2218, and IONIS-HBVRx; JNJ-3989 consists of 2 short-interfering RNA (siRNA), inhibiting the expression of both exogenous and endogenous genes, with potential for both prevention and eradication of viral infections such as HIV, HBV, and HCV, for which current therapies are insufficient [53].

Clinical studies have identified JNJ-3989 (ARO-HBV) and ARB-1740 as well-tolerated drugs that significantly reduce HBsAg levels. In a Phase IIb clinical trial, 39 participants achieved a decrease of ≥1.0 log10 IU/mL in HBsAg, and 22 patients showed a significant decrease in HBsAg during monitoring. The average reduction in HBsAg was 1.74 log10 IU/mL [54].

In the APASL 2020 study, the progression of HBV chronic hepatitis (CHB) patients undergoing treatment with JNJ-3989, JNJ-6379, and NAs was analyzed. The study lasted for 12 weeks, during which 12 patients received treatment with NAs, supplemented with a subcutaneous injection of 200 mg JNJ-3989 on days 1, 29, and 57, and oral JNJ-6379 (250 mg daily). Significant reductions in HBV RNA, HBV DNA, hepatitis B core-related antigen (HBcrAg), and HBeAg were observed [55]. Therefore, JNJ-3989 is a safe and well-tolerated antiviral, with significant reductions in viral markers and no significant adverse reaction [54].

VIR-2218 is the first RNA to utilize Enhanced Stabilization Chemistry Plus technology to increase stability. It acts on the regions of the HBV X gene shared by HBV transcripts, reducing both HBV RNA transcriptions from cccDNA and integrated DNA. In the 2021 EASL study, the majority of participants achieved the maximum decline in HBsAg levels by week 16, but after 24 weeks, a return to average HbsAg-values was observed across all treatment arms [56]. Currently, this medication is in a Phase II clinical trial.

IONIS-HBVRx is a 2’-MOE-modified oligonucleotide (ASO) that acts on HBV RNA [8]. ASO is a molecular drug that acts by inhibiting gene expression, through the specific sequence association with the target mRNA or DNA gene [57].

In one study, after 4 weeks of treatment in previously untreated CHB patients, inhibition of both HBsAg and HBV DNA was observed [57]. Currently, IONIS-HBVRx is in a Phase II clinical trial.

##### Capsid Assembly Inhibitors: GLS4 and ABI-H0731

Before DNA replication takes place, pregenomic RNA (pgRNA) and polymerase are encapsidated. Thus, capsid assembly inhibitors act by blocking the nucleocapsid formation process, affecting viral replication and replenishing cccDNA [58]. When GLS4/RTV is combined with ETV, the antiviral activity is significant, and the treatment is safe, achieving better outcomes [59].

ABI-H0731 not only blocks the encapsidation of HBV pgRNA and replication of the DNA but also prevents the formation of new cccDNA by relaxing the circular DNA that is supposed to be introduced into the nucleus [60].

In a Phase II clinical trial (ABI-H0731-201), 73 patients were recruited, including 47 with CHB and AgHBe-positive, and 26 patients with AgHBe-negative, achieving viral suppression. They were administered 300mg of ABI-H0731 daily or NAs-placebo for 24 weeks. In another study (ABI-H0731-202), 25 untreated patients with CHB and AgHBe-positive were recruited to receive ABI-H0731 in combination with ETV or ETV monotherapy, for 24 weeks [61].

The results of patients with CHB and HBeAg-positive from the two aforementioned studies were announced at the 2019 AASLD conference. In the first study ABI-H0731-201, 41% achieved HBV RNA <35 U/mL, undetectable viremia, and HBeAg < 1 U/mL. In the second study, the mean reductions in HBV DNA, HBV RNA, HBeAg, HBcrAg, and HBsAg in 22 patients were 6.1, 3.0, ≥0.6, >0.8, respectively, ≥0.4 log10 IU/mL [62]. ABI-H0731-211 was an open-label extension study that allowed patients, from the two previously-mentioned studies, to continue receiving ABI-H0731 in combination with NAs for 1 year. The selected patients for this study were those meeting the criteria for cessation of treatment, and they discontinued antiviral administration with monthly safety monitoring and recurrence surveillance. Ultimately, the study did not achieve a significant sustained virological response (SVR) rate, as 39 out of 41 participants experienced viremia increases after treatment cessation [63].

The use of ABI-H0731 in combination with NAs is safe, with few adverse reactions, and in patients with CHB treated with this combination, a significant decrease in HBV DNA and HBV RNA has been observed [62,63].

##### Inhibitor of HBsAg Secretion: REP2139

The nucleic acid polymer (NAP), REP 2139, inhibits the release of HBsAg by blocking the assembly and secretion of HBV subviral particles. Additionally, circulating HBsAg is eliminated in order to establish functional control of HBV, which persists after treatment discontinuation. The benefits of using this treatment include high rates of seroconversion and HBsAg-clearance, significant virological control, as well as functional cure for patients with HBV [64]. Currently, REP2139 is being evaluated in a Phase II clinical trial.

The complete cure of HBV is a current goal that has not been achieved yet, due to the limitations of current antiviral therapy. In recent studies, the commonly used strategy is the combination of antivirals with immunomodulators to enhance the functional cure of HBV [65,66].

### 3.5. Clinical Results with Current Antiviral Medication

In the study by Batirel A et al. [67], 195 patients with CBH were included, of which more than half (54%) underwent treatment with ETV, and only 46% received TDF. The majority of patients were male, accounting for 72%, with a mean age of 43 ± 12 years and an average duration of antiviral treatment administration of 30.2 ± 15.7 months (Table 1). AgHBe-seroconversion in AgHBe-positive patients who received ETV was 39%, and in those who received TDF, it was 24%, with a p-value of 0.2; thus, the difference was statistically insignificant. The normalization of ALT was the same in both study groups (*p* > 0.05) (Table 1).

Clinical studies have noted that the viral load is directly proportional to the risk of complications such as hepatocellular carcinoma (HCC) or liver cirrhosis [68,69]. Therefore, a primary therapeutic goal—to prevent complications and the progression of liver damage—is the sustained suppression of HBV DNA [70,71].

In the study conducted by Choi J et al. [72], an initial cohort of 24,156 patients was analyzed, where it was observed that the risk of HCC occurrence and mortality were significantly lower in patients with CBH treated with TDF compared to those treated with ETV. These conclusions were supported by another hospital-based cohort that included 2701 CBH patients, not previously treated similarly to the aforementioned cohort [72] (Table 1).

Choi J et al. [72] found that the risk of HCC occurrence is lower with the use of TDF, which could be partially explained by the viral resistance (VR) profiles of patients with TDF observed in the hospital cohort, but which are also in line with previous study results [67,73,74].

In the study conducted by Sriprayoon T et al. [75], treatment-naive patients with CBH who initiated antiviral treatment with TDF or ETV achieved viral suppression more rapidly. Additionally, a significant decrease in HBsAg was observed compared to the initial values, and among all participating patients in the study, 1.0–1.5% lost HBsAg over the course of 144 weeks (Table 1). This study identified predictive factors that could influence viral suppression through a univariate analysis. It was concluded that one of the negative predictive factors for viral suppression is advanced age, along with the initial viral load values and the positivity of HBeAg during the study. Most HBeAg-positive patients at the start of treatment presented higher initial viral loads compared to those who were HBeAg-negative. Thus, low initial viremia was one of the predictive factors for adequate viral suppression at weeks 96 and 144, with a percentage of 91% for the ETV group and 94% for those receiving TDF. HBeAg seroconversion in patients with a positive result at initiation of treatment was 27.4% and 33.7% for ETV, respectively. Both drugs demonstrated strong antiviral activity with comparable efficacy without significant adverse reactions and without renal toxicity (Table 1).

Kim YM et al. [76] monitored the virological response (HBV DNA level of less than 20 IU/mL) in HBV patients who had not received any antiviral treatment before and in patients who had previously tried another antiviral and developed resistance (antivirals such as lamivudine, adefovir). It was observed that treatment-naive patients achieved virological response much faster: 12.3 ± 10.5 months compared to 20.7 ± 27.4 months in experienced NA patients (*p* = 0.138). In this study, TDF treatment, high levels of HBeAg, low viremia, and HBe-negative status were predictive factors of HCC occurrence for multivariate analysis (Table 1).

Perrillo RP et al. [77] and Chien RN et al. [78] also studied virological response after antiviral treatment and the initial ALT level as a predictive factor of HCC occurrence. The annual rates of HCC occurrence were 1.27% for the ETV-treated group and 0.85% for those on TDF, with a *p*-value of 0.526, indicating no significant difference.

In the study conducted by Cai D et al. [79], at the end of 144 weeks, viral suppression was similar in the two groups (ETV vs. TDF; −6.6485 vs. −6.692 log10 IU/mL, *p* = 0.807), with no significant changes in biochemical and serologic response (Table 1). Out of the three hundred and twenty patients who initiated antiviral therapy, two of them ceased treatment due to adverse reactions, and five experienced severe adverse reactions. The most common adverse reactions included elevated levels of alkaline phosphatase (ALP), creatine kinase (CK), total bilirubin, leukocytes, and alanine transaminase (ALT). In addition to these, headaches or hepatic steatosis may also occur.

**Table 1:** The results of antiviral therapy in chronic HBV in clinical trials.

Author	Antiviral Treatment	Number of Patients	% Male	Mean Age ± SD (Years)	Duration of Treatment (Weeks)	% AgHBe-Positive	Initial HVB- DNA Mean ± SD (log10 IU/mL)	Initial ALT Mean ± SD (UI/mL)	%AgHBe- Sero Conversion	% Undetectable HBV-DNA	% Normalized ALT	%AgHbs- Sero conversion	%HCC Incidence	%CH Incidence
Batirel A et al. [67]	TDF	90	59	43.3 ± 12.9	27.2 ± 15.4 #	32.2	191,613 ± 198.6	116.7 ± 92.6	24	85.6	97.8	1.1	0	0
	ETV	105	78.1	42.0 ± 11.2	33.0 ± 15.4 #	34.3	220,199 ± 101.3	120.0 ± 96.6	39	85.7	99	0.9	0	0
Choi J et al. [72]	TDF	1141	60.6	48.1 ± 10.5	364	56.2	6.4	152.6 ± 303.8	19.8*	NA	44.3 ”*	NA	NA	57.2
	ETV	1560	61.9	49.2 ± 10.5		54.7	6.7	176.3 ± 314.7	20.2*	NA	44.3 ”*	NA	NA	59.9
Kim YM et al. [78]	TDF	112	62.5	49.3 ± 10.9	494	55.4	6.0 ± 1.5	67	NA	NA	NA	NA	0.8	NA
	ETV	191	60.7	47.7 ± 12.3		60.7	6.3 ± 1.2	124.5	NA	NA	NA	NA	1.2	NA
Cai D et al. [79]	TDF	157	75.8	30.7 ± 8.7	144	NA	7.5 ± 0.9	NA	32.6	91.6	86.3	NA	NA	NA
	ETV	158	76.5	30.9 ± 8.4		NA	7.6 ± 0.9	NA	34.7	86.9	87.7	NA	NA	NA
Sriprayoon T et al. [75]	TDF	200	56.5	41.2 ± 11.6	144	46	7.0 ± 1.4	76.8 ± 79.8	33.7	94 ^	86.5 ”	NA	NA	14.5
	ETV	200	76.5	41.6 ± 11.5		47.5	7.1 ± 1.5	68.1 ± 64.1	27.4	91 ^	88.5 ”	NA	NA	15.5
Our unpublished data	ETV	75	69.3	59.8 ± 11.7	271.62 ± 151.81 #	9.3	31,198,005.3 ± 111,940,457.4	70.4 ± 90.7	28.5	98.6	94.6 ”	0	4	20

Abbreviations: # mean ± SD, * at 1 year, ^ HBV DNA < 20 IU/mL, ” Choi J et al. [72]. ALT ≤ 30 IU/mL for males and ALT ≤ 19 IU/mL for females, for Sriprayoon T et al. [75]. ALT < 40 U/L, ALT < 45 U/L for our data.

Regarding the evolution of the degree of liver fibrosis under long-term treatment with NA, clinical studies have observed improvement in the degree of fibrosis and regression of advanced fibrosis [80,81,82].

The degree of fibrosis, according to the Ishak score, improved over a period of 3 and 6 years in patients treated with ETV, with 57% in the first 3 years and 88% at 6 years [83,84].

In patients treated with TDF, 87% of patients treated for 240 weeks had histological improvement in liver biopsy, and 51% had regression of fibrosis (*p* < 0.0001) [85].

The benefits of long-term antiviral therapy with NA are improvement of fibrosis, conversion of advanced fibrosis, prevention of aggravation of fibrosis, and prevention of the occurrence of liver cirrhosis, as well as decrease of the risk of occurrence of HC [70].

## 4. Discussion

The clinical studies and meta-analyses included in our analysis evaluated antiviral therapy with ETV or TDF in groups of over 50 patients with an average age over 30 years, predominantly men who received these therapies for several tens or hundreds of weeks.

These studies included HBeAg-positive and negative patients, with positive HBV DNA and initial ALT elevated above the normal value. The percentage of obtaining undetectable HBV DNA was between 85.6 and 98.6%. The percentage of seroconversion of HBeAg was between 19.8 and 39%, and of HBsAg, it was between 0 and 1.1%. The percentage of liver cirrhosis in the study groups varied between 0 and 59.9% and the percentage of liver carcinoma in these study groups was between 0 and 4%.

Gane EJ et al. evaluated the efficacy and safety of selgantolimod treatment in HBV patients already on antiviral therapy in a phase II study. They followed 48 patients who were divided into two cohorts for 24 weeks. To finally assess efficacy, a decrease in HBsAg by ≥1 log10 IU/mL from the time of initiation of treatment until the end of week 24 was followed, and at the end of the study, patients continued for an additional 24 weeks on antiviral therapy. Patients treated with a placebo (nine patients) had smaller decreases compared to those treated with selgantolimod (thirty-nine patients) at the end of week 24; 18% had significant decreases in HBsAg, and at the end of week 48, they had a percentage of 26%. Regarding adverse effects following new therapies, the most common were nausea in 46%, both vomiting and upper respiratory tract infections in 23%, and gastrointestinal disorders, which were transient and mostly mild. The effects of selgantolimod administration are dose-dependent and produce a transient increase in service cytokines, such as IFN-γ, IL-12p40, and IL-1RA, as well as promote the redistribution of subgroups of circulating immune cells. The treatment was generally well tolerated, but further studies are needed to understand the long-term effects of these new therapies [86].

Gane EJ et al. studied the pharmacokinetics and antiviral activity of a new capsid assembly modulator, JNJ-64530440, administered alone to patients with naive HBV for 4 weeks. The therapy was administered in a single dose of 750 mg per day or twice daily for 4 weeks. Following dosing, significant decreases in viraemia compared to the baseline (−3.2 vs. −3.3 log10 IU/mL, respectively) were observed; this is due to an increase in plasma concentrations after the administration of JNJ-64530440 [87].

Given the limitations of comparisons between studies, the decreases in viraemia observed in this study can be compared with other phase 1b CAMs, such as velbicovir (ABI-H0731; CAM-N), after 4 weeks of administration, with a maximum decrease of 2.8 log10 IU/mL [88]; JNJ-56136379, with a maximum decrease of 2.92 log10 IU/mL [89]; RO7049389 (CAM-A), with a maximum decrease of 3.0 log10 IU/mL [90]; and NVR 3-778 (CAM-N), with a maximum decrease of 1.97 log10 IU/mL [91].

Among the limitations of this study are the small size of the study group, the predominance of a male and white population, especially positive HBeAg, Asian patients, and the small duration of the study.

Finally, this molecule, whether administered at a single dose or two times daily, was well tolerated by patients without severe adverse reactions, with substantial results of significant decreases in HBV DNA and HBV RNA compared to the baseline [87].

Our study included 75 patients treated with entecavir between 62 and 694 weeks with a mean of 271.62, standard deviation (SD) of 151.81, and a median of 276 weeks. They were predominantly men (69.3%), aged between 29 and 82 years (average 59.82 years, SD 11.79, median 62 years). At the beginning of the treatment, there were seven patients with positive HBeAg, of which two made seroconversion. No patient had HBsAg seroconversion. The batch contains twenty patients with liver cirrhosis and three patients who developed liver carcinoma. The therapy was well tolerated, there were no interruptions or changes of therapy due to adverse reactions, and the patients were adherent to the therapy and maintained undetectable HBV DNA or with values up to 100 IU/mL.

## 5. Conclusions

From the point of view of a risk–benefit relationship, current therapies for chronic HBV infection are well tolerated, with strong antiviral adherence and low antiviral resistance. The target of the new innovative antiviral therapies is the loss of HBsAg, and the preliminary results of the clinical studies indicate good efficiency rates and a convenient profile of adverse reactions, which opens the perspective of sustained viral response in chronic HBV.

## Data Availability

Data are available upon request through the corresponding author.

## References

[1] World Health Organization (2021). Global Progress Report on HIV, Viral Hepatitis and Sexually Transmitted Infections. https://www.who.int/publications/i/item/9789240027077.

[2] Lavanchy D. (2004). Hepatitis B virus epidemiology, disease burden, treatment, and current and emerging prevention and control measures. J. Viral Hepat..

[3] Perz J.F., Armstrong G.L., Farrington L.A., Hutin Y.J., Bell B.P. (2006). The contributions of hepatitis B virus and hepatitis C virus infections to cirrhosis and primary liver cancer worldwide. J. Hepatol..

[4] Goldstein S.T., Zhou F., Hadler S.C., Bell B.P., Mast E.E., Margolis H.S. (2005). A mathematical model to estimate global hepatitis B disease burden and vaccination impact. Int. J. Epidemiol..

[5] World Health Organization (2007). Hepatitis B 2007. World Health Organization Fact Sheet 204 (Revised August 2008).

[6] De Man R.A., Heijtink R.A., Niesters H.G., Schalm S.W. (1995). New developments in antiviral therapy for chronic hepatitis B infection. Scand J. Gastroenterol. Suppl..

[7] Cornberg M., Lok A.S., Terrault N.A., Zoulim F. (2020). Faculty E-AHTEC. Guidance for design and endpoints of clinical trials in chronic hepatitis B—Report from the 2019 EASL-AASLD HBV Treatment Endpoints Conference (double dagger). J. Hepatol..

[8] Tang Y., Liang H., Zeng G., Shen S., Sun J. (2022). Advances in new antivirals for chronic hepatitis B. Chin. Med. J..

[9] Fanning G.C., Zoulim F., Hou J., Bertoletti A. (2019). Therapeutic strategies for hepatitis B virus infection: Towards a cure. Nat. Rev. Drug Discov..

[10] Naggie S., Lok A.S. (2021). New Therapeutics for Hepatitis B: The Road to Cure. Annu. Rev. Med..

[11] Terrault N.A., Lok A.S.F., McMahon B.J., McMahon B.J., Chang K.M., Hwang J.P., Jonas M.M., Brown R.S., Bzowej N.H., Wong J.B. (2018). Update on prevention, diagnosis, and treatment of chronic hepatitis B: AASLD 2018 hepatitis B guidance. Hepatology.

[12] Asselah T., Loureiro D., Boyer N., Mansouri A. (2019). Targets and future direct-acting antiviral approaches to achieve hepatitis B virus cure. Lancet Gastroenterol. Hepatol..

[13] Zhou K., Contag C., Whitaker E., Terrault N. (2019). Spontaneous loss of surface antigen among adults living with chronic hepatitis B virus infection: A systematic review and pooled meta-analyses. Lancet Gastroenterol. Hepatol..

[14] Wang G.Q., Wang F.S., Cheng J., Ren H., Zhuang H., Sun J. (2015). The guideline of prevention and treatment for chronic hepatitis B: A 2015 update. J. Clin. Hepatol..

[15] Zheng X., Wang J., Yang D. (2015). Antiviral therapy for chronic hepatitis B in China. Med. Microbiol. Immunol..

[16] Lok A.S., McMahon B.J. (2009). Chronic hepatitis B: Update 2009. Hepatology.

[17] Janssen H.L., van Zonneveld M., Senturk H., Zeuzem S., Akarca U.S., Cakaloglu Y., Simon C., So T.M., Gerken G., de Man R.A. (2005). Pegylated interferon alfa-2b alone or in combination with lamivudine for HBeAg-positive chronic hepatitis B: A randomised trial. Lancet.

[18] Marcellin P., Heathcote E.J., Buti M., Gane E., de Man R.A., Krastev Z., Germanidis G., Lee S.S., Flisiak R., Kaita K. (2008). Tenofovir disoproxil fumarate versus adefovir dipivoxil for chronic hepatitis B. N. Engl. J. Med..

[19] Association CSoIDCM, Association CSoHCM (2019). Guidelines for the prevention and treatment of chronic hepatitis B (version 2019). J. Clin. Hepatol..

[20] Chang T.T., Gish R.G., de Man R., Gadano A., Sollano J., Chao Y.C., Lok A.S., Han K.H., Goodman Z., Zhu J. (2006). A comparison of entecavir and lamivudine for HBeAg-positive chronic hepatitis B. N. Engl. J. Med..

[21] Lai C.L., Shouval D., Lok A.S., Chang T.T., Cheinquer H., Goodman Z., DeHertogh D., Wilber R., Zink R.C., Cross A. (2006). Entecavir versus lamivudine for patients with HBeAg-negative chronic hepatitis B. N. Engl. J. Med..

[22] Chang T.T., Lai C.L., Kew Yoon S., Lee S.S., Coelho H.S., Carrilho F.J., Poordad F., Halota W., Horsmans Y., Tsai N. (2010). Entecavir treatment for up to 5 years in patients with hepatitis B e antigen-positive chronic hepatitis B. Hepatology.

[23] Keating G.M. (2011). Entecavir: A review of its use in the treatment of chronic hepatitis B in patients with decompensated liver disease. Drugs.

[24] Marcellin P., Gane E.J., Flisiak R., Trinh H.N., Petersen J. (2014). Long term treatment with tenofovir disoproxil fumarate for chronic hepatitis B infection is safe and well tolerated and associated with durable virologic response with no detectable resistance: 8 year results from two phase 3 trials. Hepatology.

[25] Chan H.L.Y., Lim Y.S., Seto W.K.W., Agarwal K., Brunetto M.R., Janssen H., Căruntu F.A., Stepanova T., Tsang O.T., Yatsuhashi H. (2018). Three year efficacy and safety of tenofovir alafenamide (TAF) compared to tenofovir disoproxil fumarate (TDF) in HBeAg-negative and HBeAg-positive patients with chronic hepatitis B. Hepatology.

[26] Agarwal K., Brunetto M., Seto W.K., Lim Y.S., Fung S., Marcellin P., Ahn S.H., Izumi N., Chuang W.L., Bae H. (2018). 96 weeks treatment of tenofovir alafenamide vs. Tenofovir disoproxil fumarate for hepatitis B virus infection. J. Hepatol..

[27] Raffi F., Orkin C., Clarke A., Slama L., Gallant J., Daar E., Henry K., Santana-Bagur J., Stein D.K., Bellos N. (2017). Brief Report: Long-term (96-week) Efficacy and Safety After Switching from Tenofovir Disoproxil Fumarate (TDF) to Tenofovir Alafenamide (TAF) in HIV-infected, Virologically Suppressed Adults. J. Acquir. Immune Defic. Syndr..

[28] Lau G.K.K., Piratvisuth T., Luo K.X., Marcellin P., Thongsawat S., Cooksley G., Gane E., Fried M.W., Chow W.C., Paik S.W. (2005). Peginterferon Alfa-2a, lamivudine, and the combination for HBeAg-positive chronic hepatitis B. N. Engl. J. Med..

[29] Marcellin P., Bonino F., Lau G.K.K., Farci P., Yurdaydin C., Piratvisuth T., Jin R., Gurel S., Lu Z.M., Wu J. (2009). Sustained response of hepatitis B e antigen-negative patients 3 years after treatment with peginterferon Alfa-2a. Gastroenterology.

[30] Ono A., Suzuki F., Kawamura Y., Sezaki H., Hosaka T., Akuta N., Kobayashi M., Suzuki Y., Saitou S., Arase Y. (2012). Long-term continuous entecavir therapy in nucleos(t)ide-naïve chronic hepatitis B patients. J. Hepatol..

[31] Seto W.K., Wong D.K.H., Fung J., Huang F.Y., Lai C.L., Yuen M.F. (2013). Reduction of hepatitis B surface antigen levels and hepatitis B surface antigen seroclearance in chronic hepatitis B patients receiving 10 years of nucleoside analogue therapy. Hepatology.

[32] Tsai E. (2021). Review of Current and Potential Treatments for Chronic Hepatitis B Virus Infection. Gastroenterol. Hepatol..

[33] Baroiu L., Anghel L., Tatu A.L., Iancu A.V., Dumitru C., Leșe A., Drăgănescu M., Năstase F., Niculeț E., Fotea S. (2022). Risk of hepatitis B reactivation: From biologic therapies for psoriasis to immunosuppressive therapies for COVID-19 (Review). Exp. Ther. Med..

[34] The Romanian Protocol for Therapy of HBV Infection. https://www.formaremedicala.ro/protocolul-terapeutic-in-hepatita-cronica-si-ciroza-hepatica-cu-virus-vhb-lb01b/.

[35] WHO—HBV Vaccination. https://www.who.int/news-room/fact-sheets/detail/hepatitis-b.

[36] Chang M.H., Chen D.S. (2015). Prevention of hepatitis B. Cold Spring Harb. Perspect. Med..

[37] Halichidis S., Dumea E., Cambrea C.S. (2013). Seroclearance of Hepatitis B surface antigen after entecavir treatment. J. Gastroin-Testinal Liver Dis..

[38] Leblebicioglu H., Arama V., Zarski J.P. (2014). Predictors associated with treatment initiation and switch in a real-world chronic hepatitis B population from five European countries. J. Viral Hepat..

[39] Cambrea S.C., Arghir O.C., Rascu A., Petcu C.L. (2018). Biochemical Features of an Acute Viral Hepatitis A Outbreak. Revista de Chi-mie.

[40] Herrscher C., Roingeard P., Blanchard E. (2020). Hepatitis B Virus Entry into Cells. Cells.

[41] Urban S., Bartenschlager R., Kubitz R., Zoulim F. (2014). Strategies to inhibit entry of HBV and HDV into hepatocytes. Gastroenterology.

[42] Hong H.J., Ryu C.J., Hur H., Kim S., Oh H.K., Oh M.S., Park S.Y. (2004). In vivo neutralization of hepatitis B virus infection by an anti-preS1 humanized antibody in chimpanzees. Virology.

[43] Wi J., Jeong M.S., Hong H.J. (2017). Construction and Characterization of an Anti-Hepatitis B Virus preS1 Humanized Antibody that Binds to the Essential Receptor Binding Site. J. Microbiol. Biotechnol..

[44] Eke A.C., Eleje G.U., Eke U.A., Xia Y., Liu J. (2017). Hepatitis B immunoglobulin during pregnancy for prevention of mother-to-child transmission of hepatitis B virus. Cochrane Database Syst. Rev..

[45] Katz L.H., Paul M., Guy D.G., Tur-Kaspa R. (2010). Prevention of recurrent hepatitis B virus infection after liver transplantation: Hepatitis B immunoglobulin, antiviral drugs, or both? Systematic review and meta-analysis: Post liver transplant hepatitis B prophylaxis. Transpl. Infect. Dis..

[46] Tsounis E.P., Tourkochristou E., Mouzaki A., Triantos C. (2021). Toward a new era of hepatitis B virus therapeutics: The pursuit of a functional cure. World J. Gastroenterol..

[47] Krepstakies M., Lucifora J., Nagel C.H., Zeisel M.B., Holstermann B., Hohenberg H., Kowalski I., Gutsmann T., Baumert T.F., Brandenburg K. (2012). A new class of synthetic peptide inhibitors blocks attachment and entry of human pathogenic viruses. J. Infect. Dis..

[48] Andrä J., Howe J., Garidel P., Rössle M., Richter W., Leiva-León J., Moriyon I., Bartels R., Gutsmann T., Brandenburg K. (2007). Mechanism of interaction of optimized Limulus-derived cyclic peptides with endotoxins: Thermodynamic, biophysical and microbiological analysis. Biochem. J..

[49] Gutsmann T., Razquin-Olazarán I., Kowalski I., Kaconis Y., Howe J., Bartels R., Hornef M.W., Schürholz T., Rössle M., Sanchez-Gomez S. (2010). New antiseptic peptides to protect against endotoxin-mediated shock. Antimicrob. Agents Chemother..

[50] Tsukuda S., Watashi K., Hojima T., Isogawa M., Iwamoto M., Omagari K., Suzuki R., Aizaki H., Kojima S., Sugiyama M. (2017). A new class of hepatitis B and D virus entry inhibitors, proanthocyanidin and its analogs, that directly act on the viral large surface proteins. Hepatology.

[51] Xia Y., Stadler D., Lucifora J., Reisinger F., Webb D., Hösel M., Michler T., Wisskirchen K., Cheng X., Zhang K. (2016). Interferon-γ and Tumor Necrosis Factor-α Produced by T Cells Reduce the HBV Persistence Form, cccDNA, Without Cytolysis. Gastroenterology.

[52] Wedemeyer H., Schöneweis K., Bogomolov P.O., Voronkova N., Chulanov V., Stepanova T., Bremer B., Allweiss L., Dandri M., Burhenne J. (2019). GS-13-Final results of a multicenter, open-label phase 2 clinical trial (MYR203) to assess safety and efficacy of myrcludex B in cwith PEG-interferon alpha 2a in patients with chronic HBV/HDV co-infection. J. Hepatol..

[53] Wu J., Nandamuri K.M. (2004). Inhibition of hepatitis viral replication by siRNA. Expert. Opin. Biol. Ther..

[54] Gane E., Locarnini S., Lim T.H., Strasser S., Sievert W., Cheng W., Thompson A.J., Given B.D., Schluep T., Hamilton J. (2020). Short-term treatment with RNA interference therapy, JNJ-3989, results in sustained hepatitis B surface antigen suppression in patients with chronic hepatitis B receiving nucleos(t)ide analogue treatment. J. Hepatol..

[55] Man-Fung Y., Stephen L., Bruce G., Thomas S., James H., Michael B. (2020). First clinical experience with RNA interference [RNAi]-based triple combination therapy in chronic hepatitis B (CHB): JNJ73763989 (JNJ-3989), JNJ-56136379 (JNJ-6379) and a nucleos(t)ide analogue (NA). Hepatol. Int..

[56] Gane E., Lim Y.S., Cloutier D., Shen L., Cathcart A., Ding X. (2021). Safety and antiviral activity of VIR-2218, an X-targeting RNAi therapeutic, in participants with chronic hepatitis B infection: Week 48 follow-up results. J. Hepatol..

[57] Yuen M.F., Heo J., Jang J.W., Yoon J.H., Kweon Y.O., Park S.J., Bennett C., Kwoh T. (2020). Hepatitis B virus (HBV) surface antigen (HBsAg) inhibition with isis 505358 in chronic hepatitis B (CHB) patients on stable nucleos (t)ide analogue (NA) regimen and in NA-naive CHB patients: Phase 2a, randomized, double-blind, placebo-controlled study. J. Hepatol..

[58] Huang A.L., Yuan Z.H., Nan Y.M., Yang D.L., Guo J.T., Li W.H. (2020). Clinical cure strategies for hepatitis B: Direct-acting antiviral drugs. Chin. J. Hepatol..

[59] Zhang M., Zhang J., Tan Y., Xin Y., Gao H., Zheng S., Yi Y., Zhang J., Wu C., Zhao Y. (2020). Efficacy and safety of GLS4/ritonavir combined with entecavir in HBeAg-positive patients with chronic hepatitis B: Interim results from phase 2b, multi-center study. J. Hepatol..

[60] Huang Q., Cai D., Yan R., Li L., Zong Y., Guo L., Mercier A., Zhou Y., Tang A., Henne K. (2020). Preclinical profile and characterization of the hepatitis B virus core protein inhibitor ABI-H0731. Antimicrob. Agents Chemother..

[61] Ma X., Lalezari J., Nguyen T., Bae H., Schiff E.R., Fung S., Yuen M., Hassanein T., Hann H., Elkhashab M. (2019). Agarwal. Interim safety and efficacy results of the ABI-H0731 phase 2a program exploring the combination of ABI-H0731 with Nuc therapy in treatment-naive and treatment-suppressed chronic hepatitis B patients. J. Hepatol..

[62] Yuen M.F., Agarwal K., Ma X.L., Nguyen T., Schiff E.R., Hann H.W., Dieterich D., Nahass R., Park J., Chan S. (2020). Antiviral activity and safety of the hepatitis B core inhibitor ABI-H0731 administered with a nucleos(t)ide reverse transcriptase inhibitor in patients with HBeAg-positive chronic hepatitis B infection in a long-term extension study. J. Hepatol..

[63] Sulkowski M.S., Agarwal K., Fung S.K., Yuen R.M.F., Ma X.L., Lalezari J.P. (2019). Continued therapy with ABI-H0731+NRTI results in sequential reduction/loss of HBV DNA, HBV RNA, HBeAg, HBcrAg and HBsAg in HBeAg positive patients. Hepatology.

[64] Bazinet M., Pantea V., Cebotarescu V., Cojuhari L., Jimbei P., Albrecht J., Schmid P., Le Gal F., Gordien E., Kraw-czyk A. (2017). Safety and efficacy of REP 2139 and pegylated interferon alfa-2a for treatment-naive patients with chronic hepatitis B virus and hepatitis D virus co-infection (REP 301 and REP301-LTF): A non-randomised, open-label, phase 2 trial. Lancet Gastroenterol. Hepatol..

[65] Ning Q., Wu D., Wang G.Q., Ren H., Gao Z.L., Hu P., Han M.-F., Wang Y., Zhang W.-H., Lu F.-M. (2019). Roadmap to functional cure of chronic hepatitis B: An expert consensus. J. Viral Hepat..

[66] Martinez M.G., Villeret F., Testoni B., Zoulim F. (2020). Can we cure hepatitis B virus with novel direct-acting antivirals?. Liver Int..

[67] Batirel A., Guclu E., Arslan F., Kocak F., Karabay O., Ozer S., Turanli M., Mert A. (2014). Comparable efficacy of tenofovir versus entecavir and predictors of response in treatment-naïve patients with chronic hepatitis B: A multicenter real-life study. Int. J. Infect. Dis..

[68] Iloeje U.H., Yang H.I., Su J., Jen C.L., You S.L., Chen C.J. (2006). Predicting cirrhosis risk based on the level of circulating hepatitis B viral load. Gastroenterology.

[69] Chen C.J., Yang H.I., Su J., Jen C.L., You S.L., Lu S.N., Huang G.T., Iloeje U.H. (2006). Risk of hepatocellular carcinoma across a biological gradient of serum hepatitis B virus DNA level. JAMA.

[70] Liaw Y.F. (2013). Impact of therapy on the outcome of chronic hepatitis B. Liver Int..

[71] Abu-Amara M., Feld J.J. (2013). Does antiviral therapy for chronic hepatitis B reduce the risk of hepatocellular carcinoma?. Semin Liver Dis..

[72] Choi J., Kim H.J., Lee J., Cho S., Ko M.J., Lim Y.S. (2019). Risk of Hepatocellular Carcinoma in Patients Treated with Entecavir vs. Tenofovir for Chronic Hepatitis B: A Korean Nationwide Cohort Study. JAMA Oncol..

[73] Zuo S.R., Zuo X.C., Wang C.J., Ma Y.T., Zhang H.Y., Li Z.J., Song L.Y., Deng Z.Z., Liu S.K. (2015). A meta-analysis comparing the efficacy of entecavir and tenofovir for the treatment of chronic hepatitis B infection. J. Clin. Pharmacol..

[74] Woo G., Tomlinson G., Nishikawa Y., Kowgier M., Sherman M., Wong D.K., Pham B., Ungar W.J., Einarson T.R., Heathcote E.J. (2010). Tenofovir and entecavir are the most effective antiviral agents for chronic hepatitis B: A systematic review and Bayesian meta-analyses. Gastroenterology.

[75] Sriprayoon T., Mahidol C., Ungtrakul T., Chun-On P., Soonklang K., Pongpun W., Laohapand C., Dechma J., Pothijaroen C., Auewarakul C. (2017). Efficacy and safety of entecavir versus tenofovir treatment in chronic hepatitis B patients: A randomized controlled trial. Hepatol. Res..

[76] Kim Y.M., Shin H.P., Lee J.I., Joo K.R., Cha J.M., Jeon J.W., Yoon J.Y., Kwak M.S. (2018). Real-world single-center experience with entecavir and tenofovir disoproxil fumarate in treatment-naïve and experienced patients with chronic hepatitis B. Saudi J. Gastroenterol..

[77] Perrillo R.P., Lai C.L., Liaw Y.F., Dienstag J.L., Schiff E.R., Schalm S.W., Heathcote E.J., Brown N.A., Atkins M., Woessner M. (2002). Predictors of HBeAg loss after lamivudine treatment for chronic hepatitis B. Hepatology.

[78] Chien R.N., Liaw Y.F., Atkins M. (1999). Pretherapy alanine transaminase level as a determinant for hepatitis B e antigen seroconversion during lamivudine therapy in patients with chronic hepatitis B. Asian Hepatitis Lamivudine Trial Group. Hepatology.

[79] Cai D., Pan C., Yu W., Dang S., Li J., Wu S., Jiang N., Wang M., Zhang Z., Lin F. (2019). Comparison of the long-term efficacy of tenofovir and entecavir in nucleos(t)ide analogue-naïve HBeAg-positive patients with chronic hepatitis B: A large, multicentre, randomized controlled trials. Medicine.

[80] Dienstag J.L., Goldin R.D., Heathcote E.J., Hann H.W., Woessner M., Stephenson S.L., Gardner S., Gray D.F., Schiff E.R. (2003). Histological outcome during long-term lamivudine therapy. Gastroenterology.

[81] Hadziyannis S.J., Tassopoulos N.C., Heathcote E.J., Chang T.T., Kitis G., Rizzetto M., Marcellin P., Lim S.G., Goodman Z., Ma J. (2006). Long-term therapy with adefovir dipivoxil for HBeAg-negative chronic hepatitis B for up to 5 years. Gastroenterology.

[82] Marcellin P., Chang T.T., Lim S.G., Tong M.J., Sievert W., Shiffman M.L., Jeffers L., Goodman Z., Wulfsohn M.S., Xiong S. (2008). Long-term efficacy and safety of adefovir dipivoxil for the treatment of hepatitis B e antigen-positive chronic hepatitis B. Hepatology.

[83] Yokosuka O., Takaguchi K., Fujioka S., Shindo M., Chayama K., Kobashi H., Hayashi N., Sato C., Kiyosawa K., Tanikawa K. (2010). Long-term use of entecavir in nucleoside-naïve Japanese patients with chronic hepatitis B infection. J. Hepatol..

[84] Chang T.T., Liaw Y.F., Wu S.S., Schiff E., Han K.H., Lai C.L., Safadi R., Lee S.S., Halota W., Goodman Z. (2010). Long-term entecavir therapy results in the reversal of fibrosis/cirrhosis and continued histological improvement in patients with chronic hepatitis B. Hepatology.

[85] Marcellin P., Gane E., Buti M., Afdhal N., Sievert W., Jacobson I.M., Washington M.K., Germanidis G., Flaherty J.F., Aguilar Schall R. (2012). Regression of cirrhosis during tenofovir disoproxil fumarate treatment for chronic hepatitis B. Lancet.

[86] Gane E.J., Dunbar P.R., Brooks A.E., Zhang F., Chen D., Wallin J.J., van Buuren N., Arora P., Fletcher S.P., Tan S.K. (2023). Safety and efficacy of the oral TLR8 agonist selgantolimod in individuals with chronic hepatitis B under viral suppression. J. Hepatol..

[87] Gane E.J., Schwabe C., Berliba E., Tangkijvanich P., Jucov A., Ghicavii N., Verbinnen T., Lenz O., Talloen W., Kakuda T.N. (2022). Safety, antiviral activity and pharmacokin8etics of JNJ-64530440, a novel capsid assembly modulator, as 4 week monotherapy in treatment-naive patients with chronic hepatitis B virus infection. J. Antimicrob. Chemother..

[88] Yuen M.F., Agarwal K., Gane E.J., Schwabe C., Ahn S.H., Kim D.J., Lim Y.S., Cheng W., Sievert W., Visvanathan K. (2020). Safety, pharmacokinetics, and antiviral effects of ABI-H0731, a hepatitis B virus core inhibitor: A randomised, placebo-controlled phase 1 trial. Lancet Gastroenterol. Hepatol..

[89] Zoulim F., Lenz O., Vandenbossche J.J., Talloen W., Verbinnen T., Moscalu I., Streinu-Cercel A., Bourgeois S., Buti M., Crespo J. (2020). JNJ-56136379, an HBV capsid assembly modulator, is well-tolerated and has antiviral activity in a phase 1 study of patients with chronic infection. Gastroenterology.

[90] Gane E., Liu A., Yuen M. (2018). Fetal RO7049389, a core protein allosteric modulator, demonstrates robust anti-HBV activity in chronic hepatitis B patients and is safe and well tolerated. J. Hepatol..

[91] Yuen M.F., Gane E., Kim D.J., Weilert F., Yuen Chan H.L., Lalezari J., Hwang S.G., Nguyen T., Flores O., Hartman G. (2019). Antiviral activity, safety, and pharmacokinetics of capsid assembly modulator NVR 3-778 in patients with chronic HBV infection. Gastroenterology.

