# Correction: Ignat et al. Antiviral Therapy of Chronic Hepatitis B Virus between Present and Future. *J. Clin. Med.* 2024, *13*, 2055

**DOI:** 10.3390/jcm15082970

**Published:** 2026-04-14

**Authors:** Mariana Daniela Ignat, Alexia Anastasia Stefania Balta, Raisa Eloise Barbu, Miruna Luminita Draganescu, Luiza Nechita, Doina Carina Voinescu, Aurel Nechita, Ioana Anca Stefanopol, Camelia Busila, Liliana Baroiu

**Affiliations:** 1 Doctoral School of Biomedical Sciences, ‘Dunarea de Jos’ University, 800008 Galati, Romania; mariana_daniela52@yahoo.com (M.D.I.); raisauibariu@gmail.com (R.E.B.); 2 Clinical Medical Department, Faculty of Medicine and Pharmacy, ‘Dunarea de Jos’ University, 800008 Galati, Romania; miruna.draganescu@ugal.ro (M.L.D.); nechitaluiza2012@yahoo.com (L.N.); doina.voinescu@ugal.ro (D.C.V.); aurel.nechita@ugal.ro (A.N.); camelia.busila@ugal.ro (C.B.); liliana.baroiu@ugal.ro (L.B.); 3 ‘Sf. Cuv. Parascheva’ Clinical Hospital of Infectious Diseases, 800179 Galati, Romania; 4 ‘Sf. Apostol Andrei’ Clinical Emergency County Hospital, 800578 Galati, Romania; 5 ‘Sf. Ioan’ Clinical Hospital for Children, 800487 Galati, Romania; anca.stefanopol@ugal.ro; 6 Clinical Surgical Department, Faculty of Medicine and Pharmacy, ‘Dunarea de Jos’ University, 800008 Galati, Romania

**References**

With this correction, references 20–22 from the original publication [1] have been removed. The three references in question were initially included to provide a broader conceptual or methodological context during the early development of the manuscript. However, as the manuscript was revised and its focus became more clearly defined and clinically oriented, these sources no longer played a significant role in supporting the final structure, analysis, or conclusions of the paper. We acknowledge that, in the current form, they may be considered peripheral, and we fully agree with their removal in accordance with editorial recommendations and COPE standards. The authors state that the scientific conclusions are unaffected. This correction was approved by the Academic Editor. The original publication has also been updated.
